# Consensus Report From the Miami Liver Proton Therapy Conference

**DOI:** 10.3389/fonc.2019.00457

**Published:** 2019-05-31

**Authors:** Michael D. Chuong, Adeel Kaiser, Fazal Khan, Parag Parikh, Edgar Ben-Josef, Christopher Crane, Thomas Brunner, Toshiyuki Okumura, Niek Schreuder, Søren M. Bentzen, Alonso Gutierrez, Alejandra Mendez Romero, Sang Min Yoon, Navesh Sharma, Tae Hyun Kim, Kazushi Kishi, Fred Moeslein, Sarah Hoffe, Tracey Schefter, Steven Hanish, Marta Scorsetti, Smith Apisarnthanarax

**Affiliations:** ^1^Baptist Hospital of Miami, Miami Cancer Institute, Miami, FL, United States; ^2^University of Maryland Medical Center, Baltimore, MD, United States; ^3^Henry Ford Health System, Detroit, MI, United States; ^4^Department of Radiation Oncology, University of Pennsylvania, Philadelphia, PA, United States; ^5^Department of Radiation Oncology, Memorial Sloan Kettering Cancer Center, New York, NY, United States; ^6^Freiburg University Medical Center, Freiburg, Germany; ^7^Department of Radiation Oncology, University of Tsukuba, Tsukuba, Japan; ^8^Provision CARES Proton Therapy, Knoxville, TN, United States; ^9^Erasmus Medical Center, Erasmus University Rotterdam, Rotterdam, Netherlands; ^10^Holland Proton Treatment Center, Rotterdam, Netherlands; ^11^Asan Medical Center, University of Ulsan College of Medicine, Seoul, South Korea; ^12^Department of Radiation Oncology, Pennsylvania State University, University Park, PA, United States; ^13^National Cancer Center, Goyang-si, South Korea; ^14^Hokkaido Hospital, Sapporo, Japan; ^15^Sarasota Memorial Hospital, Sarasota, FL, United States; ^16^Moffitt Cancer Center, Tampa, FL, United States; ^17^University of Colorado Anschutz Medical Campus, Aurora, CO, United States; ^18^Department of Radiation Oncology, Humanitas University, Rozzano, Italy; ^19^Department of Radiation Oncology, University of Washington, Seattle, WA, United States

**Keywords:** proton therapy, liver cancer, hepatocellular carcinoma (HCC), cholangiocarcinoma (CC), liver metastases

## Abstract

An international group of 22 liver cancer experts from 18 institutions met in Miami, Florida to discuss the optimal utilization of proton beam therapy (PBT) for primary and metastatic liver cancer. There was consensus that PBT may be preferred for liver cancer patients expected to have a suboptimal therapeutic ratio from XRT, but that PBT should not be preferred for all patients. Various clinical scenarios demonstrating appropriateness of PBT vs. XRT were reviewed.

Radiation therapy (RT) for liver cancer has become increasingly utilized as technological advancements have permitted highly conformal delivery of even ablative doses. Although RT is most commonly delivered using photons (x-rays), protons may also be considered.

In contrast to an x-ray beam, there is little to no exit dose distal to the target in a proton beam, thereby reducing low and moderate doses to normal organs. Although the clinical benefit of proton beam therapy (PBT) over x-ray therapy (XRT) has been proposed for some liver cancer patients, there is a lack of consensus to guide decision making regarding utilization of PBT. Important clinical considerations for PBT selection, however, have become better understood in recent years (1).

In January 2018, a group of 22 experts from 18 institutions across North America, Europe, and Asia congregated in Miami, Florida to discuss the role of PBT for liver cancer. Participants included thought leaders from radiation oncology, medical physics, interventional radiology, surgical oncology, and biostatistics. With the intent of conducting a balanced discussion, approximately half had PBT experience while the remainder purposefully did not. The conference goals included: (1) clarifying the role of PBT vs. XRT for liver cancer; (2) reaching consensus about clinical scenarios for which PBT provides the most significant benefit vs. XRT for liver cancer patients; and (3) identifying barriers to broader adoption of PBT for liver cancer. To address each of these goals the conference included interactive presentations, treatment plan comparisons, and surveys. The same survey questions were asked just prior to and then again immediately upon conclusion of the conference using an anonymous polling system (Table 1).

**Table 1:** Pre- and post-conference participant survey results.

**All survey questions**	**Pre-conference (%)**			**Post-conference (%)**		
**General and proton specific**	**Yes**	**No**	**Not sure**	**Yes**	**No**	**Not sure**
Is radiation therapy an effective treatment for liver cancers?	100	0	0	100	0	0
Would you consider access to proton therapy a benefit to your liver cancer program?	87	7	6	100	0	0
Should proton therapy be recommended for all liver cancer patients in place of x-ray therapy?	7	80	13	15	85	0
It is reasonable to consider proton therapy for the treatment of unresectable non-metastatic cholangiocarcinoma?	94	0	6	93	0	7
Proton therapy should be considered to treat liver metastases in patients with liver-only or liver-dominant disease	72	17	11	71	22	7
Radioembolization or chemoembolization should be preferred over proton therapy for management of some patients with extensive multifocal liver cancers?	67	33	0	86	15	7
Is the complexity of proton therapy treatment planning a barrier to proton therapy adoption?	29	65	6	21	65	14
Is respiratory motion a major obstacle for broad adoption of proton therapy for liver cancers?	33	50	17	21	65	14
Is proton therapy range uncertainty a major obstacle for broad adoption of proton therapy for liver cancers?	22	61	17	14	86	0
Is the lack of adequate image guidance a major obstacle for the broad adoption of proton therapy for liver cancers?	6	50	44	50	43	7
Is the cost of proton therapy a barrier to broad adoption?	94	6	0	100	0	0
Randomized data should be required for broad adoption of proton therapy for liver cancer patients	41	59	0	36	64	0

## Normal Liver Tolerance

The participants established that a primary rationale for PBT is sparing uninvolved liver. As a parallel functioning organ, the liver is tolerant of high dose delivered to a limited volume, presuming adequate sparing of uninvolved liver and appropriate baseline liver function (2). Minimizing both mean liver dose (MLD) and the volume of uninvolved liver receiving at least low dose should be of extreme importance for any liver-directed RT. This is underscored by the endorsement that prescription dose reduction be considered, as needed, to achieve appropriately low liver dose while still aiming to prescribe the highest possible safe dose. All agreed that PBT should be considered if MLD and low dose liver constraints cannot be achieved with XRT.

There was broad agreement that a certain absolute reduction in normal liver dose would not have the same clinical significance for all patients because the probability of radiation-induced liver disease (RILD) is also related to various clinical factors like baseline liver function. For example, a small reduction in MLD with PBT may be clinically meaningful for a patient with Child Pugh (CP)-B8 cirrhosis whereas it is less likely to be for a patient with CP-A5 cirrhosis. While normal tissue complication probability (NTCP) models are not routinely used in the management of liver cancer patients, it was agreed that such modeling could help considerably in prioritizing patients for PBT.

## Role of Radiation Therapy for Liver Cancer

There was unanimous agreement that both XRT and PBT are not experimental for treating primary or metastatic liver cancer, and that both are effective in achieving high rates of local control with acceptable toxicity.

On the pre-conference survey the overwhelming majority indicated that access to PBT would benefit their institution's liver cancer program based predominantly on the recognition of unsatisfactory clinical outcomes after XRT in some patients, particularly those with HCC and unfavorable baseline characteristics such as large tumor and suboptimal baseline liver function. All participants on the post-conference survey responded that PBT would benefit at least some liver patients at their institution, largely due to better understanding of how to overcome perceived barriers to liver PBT (e.g., target motion, range uncertainty) and the application of PBT for selected patients with non-HCC liver cancer.

There was an overarching belief that reduced normal organ dose achieved with PBT is not clinically relevant for all liver cancer patients, and that careful consideration should be given to which patients should be prioritized for PBT.

## Clinical Decision Making and Patient Prioritization for PBT

The majority of the conference was devoted to reviewing clinical factors that indicate a high probability of benefit from PBT. Conversation focused on patients with hepatocellular carcinoma (HCC), among whom hypofractionated PBT has been extensively evaluated with remarkable long-term outcomes (3).

Hypofractionated XRT outcomes for HCC have also been excellent, especially for patients with well-compensated cirrhosis (e.g., CP-A), smaller tumor size (e.g., ≤ 3–5 cm), limited number of tumors (e.g., 1–3), and no prior liver RT (4).

There was consensus that PBT should be more strongly considered for HCC patients with the following:

- At least CP-B cirrhosis
- High tumor-to-liver ratio
- Larger tumor size
- Smaller uninvolved liver volume
- Higher number of tumors
- Prior RT to the liver.

After much discussion it was determined that consensus could not be reached on absolute threshold criteria for these based on the published literature, and that best clinical judgment should be used until data are published to provide more objective guidance. Moreover, there was uniform agreement that the presence of multiple factors for RILD would further strengthen the rationale for PBT (1).

The appropriateness of treating CP-B cirrhosis patients with XRT was next debated. Some felt strongly that XRT is feasible for CP-B patients if using an individualized adaptive strategy (5) although most indicated that they do not routinely offer XRT to all CP-B patients because of concerns about potentially severe toxicity (6, 7).

**Figure 1:**
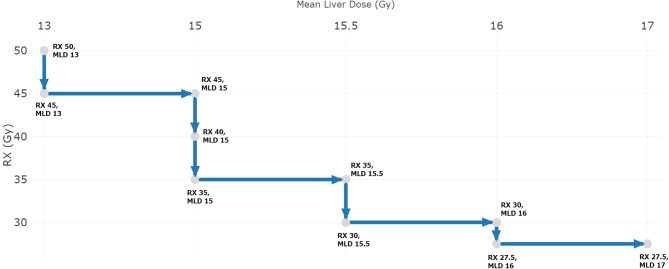
Prescription de-escalation based on achieved mean liver dose (MLD) as per RTOG 1112.

**Figure 2:**
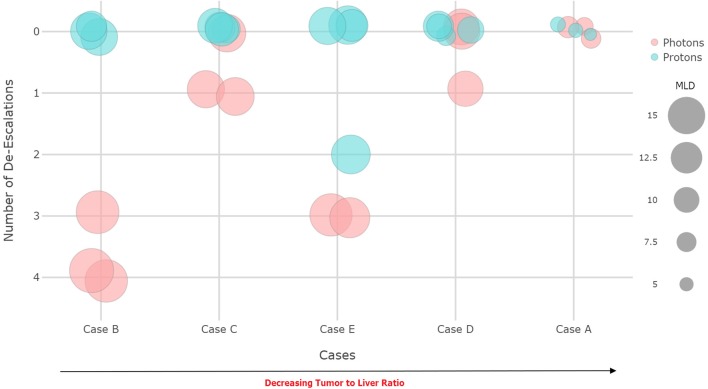
Achieved prescription dose and mean liver dose (MLD) for x-ray vs. proton treatment plans.

To illustrate what dosimetric differences can be achieved in various scenarios (Table 2), PBT and XRT plans were created for 5 cases (A-E) by institutions represented at the conference according to the Radiation Therapy Oncology Group (RTOG) 1112 protocol that requires a prescription of 50 Gy in 5 fractions based on the achievable MLD, with tiered de-escalation as needed to satisfy MLD constraints (Figure 1). The results were blinded until convening in Miami. As expected, both PBT and XRT plans were able to achieve excellent liver sparing for the smallest tumor (case A), and there was complete agreement that XRT should be preferred unless in the context of at least CP-B cirrhosis. The appropriateness of PBT was amplified with increasing tumor-to-liver ratio compared to either tumor size or uninvolved liver volume alone (Figure 2). The highest MLD and the most extensive prescription de-escalations in XRT plans occurred for case B, which did not have the largest tumor volume although featured the smallest uninvolved liver volume and highest tumor-to-liver ratio. Assuming well-compensated cirrhosis most considered XRT to be appropriate for cases C and D. Finally, while case E could be treated with PBT nearly all believed that other liver-directed therapies may be more appropriate. To emphasize that point, the participant surveys indicated that most believed radioembolization or chemoembolization to be preferred for at least some patients with extensive liver tumor burden, particularly those with numerous bilobar lesions that could not be appropriately treated with either PBT or XRT while meeting normal liver and target volume constraints.

**Table 2:** Tumor and liver characteristics of 5 cases planned with X-ray therapy (XRT) and proton beam therapy (PBT).

**Case**	**Involved lobe(s)**	**Involved segment(s)**	**Total number of tumors**	**Largest tumor diameter (cm)**	**Total tumor volume (cc)**	**Total uninvolved liver volume (cc)**	**Tumor-to-liver ratio x 100**
A	Right	8	1	5	40.7	1876.2	2.2
B	Right	6, 7, 8	1	10	216.5	1239.7	17.5
C	Right	5, 6	1	13	365.8	2453	14.9
	+ main PVTT	+ main PVTT	+ main PVTT				
D	Right	6, 7	3	3, 4, 8	89.3	2722	3.3
E	Left	2, 3, 4	5	Left Lobe: 4, 8	120	2697	4.5
	Right	6		Right Lobe: 3, 4, 8			

*PVTT, portal vein tumor thrombus*.

The participants believed that treatment planning comparisons should be considered to assist in clinical decision making between PBT and XRT although should not be required when the expected benefit from PBT is especially high.

## PBT for Cholangiocarcinoma and Liver Metastases

As opposed to HCC patients in which PBT is primarily intended to reduce the probability of liver dysfunction vs. XRT, emerging data suggest that PBT may be beneficial for selected patients with unresectable intrahepatic cholangiocarcinoma (IHC) or metastatic liver disease with the rationale being that tumor dose escalation to an ablative level may not be achievable with XRT while also respecting normal liver constraints for some patients (e.g., larger and/or numerous lesions) (8, 9). The importance of achieving an ablative tumor dose, which improves tumor control and even potentially overall survival compared to lower doses, has been demonstrated in numerous publications (10, 11).

Based on these data, most participants (70%) believed that PBT is reasonable to consider for selected patients with liver metastasis while an even larger majority (93%) believed that it is reasonable to treat unresectable non-metastatic IHC. Still, all patients should be discussed in a multidisciplinary manner with respect to all potential management options. Furthermore, it was recommended that PBT be considered in the context of potentially curative treatment for liver-only or liver-dominant disease. There was strong support for enrolling such patients to clinical trials whenever available to better define patient subsets that achieve the most meaningful clinical benefit.

## Barriers to PBT Utilization

The cost of PBT was considered by nearly all as one of the most restrictive and significant barriers to expanding PBT utilization for liver cancer. Frequent challenges with insurance approval for liver PBT were also mentioned despite the inclusion of HCC as a Group 1 indication (highest recommendation) in the ASTRO Model Policy for Proton Beam Therapy (12).

On the pre-conference survey over 40% indicated that they were not sure whether image guidance was a barrier to liver PBT, most likely reflecting that approximately 50% of participants had not treated patients with PBT and were unfamiliar with image guidance capabilities at most PBT centers. During the conference some voiced concern that PBT image guidance has historically been subpar when compared to XRT image guidance capabilities. However, there was strong sense of confidence especially among those familiar with PBT that PBT imaging techniques are currently improving including through more widespread availability of high-quality cone-beam computed tomography (CT) scans.

Commonly perceived barriers related to treatment planning were surprisingly not of concern to most. Although there are potentially severe consequences due to the interplay effect when treating moving targets, almost two-thirds felt that respiratory motion was not a major obstacle even if using pencil beam scanning (compared to passive scattering, which is more robust). Treating moving targets was considered feasible if employing effective treatment planning strategies (e.g., repainting, 4-dimensional robust optimization, robust beam angle selection, increased fractionation) combined with motion management techniques. It was noted that such strategies have been employed for decades with excellent long-term outcomes (13). Still, it should be noted that the majority of published clinical outcomes have been achieved with passive scattering and that additional study is needed with respect to pencil beam scanning. The vast majority (86%) similarly did not feel that distal range uncertainty restricts high quality liver PBT if using appropriate treatment planning techniques.

Lastly, nearly two-thirds believed that randomized data should not be an absolute requirement to justify PBT for liver cancer. Still, many agreed that randomized trials should be pursued, especially to improve our understanding of which patient subgroups should be prioritized for PBT. In the meantime, the extensive published observational data for liver cancer should be considered sufficient to justify recommending PBT.

## Conclusions

The Miami conference successfully brought together a diverse international group of experts who reached consensus that PBT is expected to dramatically improve clinical outcomes for some, but not all liver cancer patients compared to XRT. Future studies should focus on identifying which patient subgroups achieve the greatest clinical advantage from PBT to guide treatment decision making.

## Data Availability

All datasets for this study are included in the manuscript and the supplementary files.

## Author Contributions

MC contributed conception and design of the manuscript. MC, AK, and FK wrote the first draft of the manuscript. All authors contributed to manuscript revision, read, and approved the submitted version.

### Conflict of Interest Statement

This manuscript describes the outcomes of a working group meeting that was facilitated by travel support from an educational grant by IBA. IBA was not permitted to provide input on the meeting design, content, or group discussion, nor did IBA have any influence on the writing of the manuscript. The authors declare that the research was conducted in the absence of any commercial or financial relationships that could be construed as a potential conflict of interest.
